# Role and Variation of the Amount and Composition of Glomalin in Soil Properties in Farmland and Adjacent Plantations with Reference to a Primary Forest in North-Eastern China

**DOI:** 10.1371/journal.pone.0139623

**Published:** 2015-10-02

**Authors:** Qiong Wang, Wenjie Wang, Xingyuan He, Wentian Zhang, Kaishan Song, Shijie Han

**Affiliations:** 1 Key Laboratory of Forest Plant Ecology, Northeast Forestry University, Harbin, China; 2 Urban Forests and Wetlands Research Group, Key Laboratory of Wetland Ecology and Environment, Northeast Institute of Geography and Agricultural Ecology, Chinese Academy of Sciences, Changchun, China; 3 Research Institute of Forest Ecology and Forestry Ecological Engineering, Institute of Applied Ecology, Chinese Academy of Sciences, Shenyang, China; USDA Forest Service, UNITED STATES

## Abstract

The glycoprotein known as glomalin-related soil protein (GRSP) is abundantly produced on the hyphae and spores of arbuscular mycorrhizal fungi (AMF) in soil and roots. Few studies have focused on its amount, composition and associations with soil properties and possible land-use influences, although the data hints at soil rehabilitation. By choosing a primary forest soil as a non-degraded reference, it is possible to explore whether afforestation can improve degraded farmland soil by altering GRSP. In this paper, close correlations were found between various soil properties (soil organic carbon, nitrogen, pH, electrical conductivity (EC), and bulk density) and the GRSP amount, between various soil properties and GRSP composition (main functional groups, fluorescent substances, and elements). Afforestation on farmland decreased the EC and bulk density (p < 0.05). The primary forest had a 2.35–2.56-fold higher GRSP amount than those in the plantation forest and farmland, and GRSP composition (tryptophan-like and fulvic acid-like fluorescence; functional groups of C–H, C–O, and O–H; elements of Al, O, Si, C, Ca, and N) in primary forest differed from those in plantation forest and farmland (p < 0.05). However, no evident differences in GRSP amount and composition were observed between the farmland and the plantation forest. Our finding highlights that 30 years poplar afforestation on degraded farmland is not enough to change GRSP-related properties. A longer period of afforestation with close-to-nature managements may favor the AMF-related underground recovery processes.

## Introduction

Land-use changes are effective methods for soil rehabilitation, and many practices have been implemented for degraded soil recovery [1, 2]. Returning Farmland to Forest (RFTF), also referred to as the Grain for Green program, is an important environmental policy in China for rehabilitating ecological problems that include soil erosion [2, 3]. Poplar forest in north-eastern (NE) China extends into more than 90% of all farmland area (4.23 Mha) as shelterbelts for protecting farmland; these sites must be reforested after harvest due to the initiation in 1978 of national regulations (http://news.xinhuanet.com/video/2013-08/11/c_125150353.htm). More than 30 years of afforestation on farmland and well-matched paired samples (poplar plantation forests and farmland) make poplar plantation forest a good example for the study of the effects of afforestation of degraded farmland. In NE China, the climax vegetation is Korean pine-broadleaved forest, and because the loam is of good quality and without degradation, these forests can usually be used as a control in comparisons of soil degradation [4]. Comparisons of various soil properties between farmland, afforested plantation, and natural primary forest may benefit the evaluation of the effects of RFTF on soil and may also yield implications for RFTF practices, such as durations and measures need to improve the efficiency of land-use policy [1, 5].

The glycoprotein known as glomalin-related soil protein (GRSP) is abundantly produced on hyphae and spores of arbuscular mycorrhizal fungi (AMF) in soil and in roots [6, 7]. AMF and the typical secretion compounds of GRSP are well known for maintaining terrestrial ecosystems [8, 9] because they contribute to the growth of plants, influence the compositions of plant communities, and participate in various soil processes [10, 11]. GRSP could increase the stability of the soil organic carbon (SOC) pool, and its adhesive function could promote the formation of soil aggregates and improve soil quality [12]. Recent advances have found that GRSP is a mixture of many compounds, and some of these compounds are not related to AMF. For example, Schindler et al. [13] reported on the carbon content of GRSP and found differences in the aromatic carboxyl, which showed similar nuclear magnetic resonance (NMR) spectra to humic acid. Gillespie et al. [14] observed that GRSP is a rich mixture of proteinaceous, humic, lipidic, and inorganic substances and contains a consortium of proteins along with many impurities. Wang et al. [15] reported that GRSP mainly consists of stretching O–H, N–H, C–H, C = O, COO–, C–O, and Si–O–Si and bending of C–H and O–H groups, with a characteristic X-ray diffraction peak at 2*θ* = 19.8° and 129.30 nm grain size, as well as 1.08% low crystallinity. Some heavy metals, e.g. Cr and Cu, can also be stored in GRSP [16, 17], whereas the absorption of Al in GRSP can change the fluorescent density of GRSP [18]. Many of the referenced studies have tried to define the role of GRSP amount in soil carbon sequestration, fertility recovery, and degraded soil rehabilitation [19–22]. However, GRSP compositional variation and function during land-use changes have seldom been studied to date, although these data are important for a functional understanding of AMF in soil maintenance [15].

New techniques have advanced the determination of the compositional characteristics of GRSP. In the 3-D fluorescent spectrum, parallel factor (PARAFAC) analysis of the Excitation-Emission Matrix (EEM) spectra has been used to identify fluorescent organic matter in marine environments [23], red tide algae [24], vitamins [25], and green tea [26] based on fluorescence peak location and intensity. Owing to this ability to identify the complex nature of dissolved organic matter [27, 28], PARAFAC analysis has been used to effectively model EEM data sets [29] and decomposed fluorescence EEMs into independent groups of fluorescent components [30, 31]. X-ray photoelectron spectroscopy (XPS) is a surface-sensitive quantitative spectroscopic technique [32, 33]. A number of XPS protein adsorption studies used freeze-dried sample preparation and investigated the role of substrate chemistry in the distribution and orientation of protein films [34, 35]. Infrared spectroscopy (IR) made it possible to characterize the compositional differences in a more detail way, and this technique works well with other recent advances in methodology for determining GRSP composition functionality and complexity [13, 15]. In future research, the techniques of PARAFAC analysis of EEM spectra, XPS, and IR offer hope for achieving a better understanding of the GRSP compositional changes that occur during soil degradation or the recovery process.

The objectives of this study are to answer the following questions: 1) What are the possible GRSP compositional characteristics from the techniques of PARAFAC analysis of 3-D EEM spectra, XPS, and IR? 2) What are the possible relations between GRSP (amount and composition) and soil properties? 3) Whether more than 30 years of growth of poplar afforestation can improve degraded farmland soil quality by altering GRSP? And what are the possible implications of RFTF practices for soil rehabilitation of locally degraded soils in China?

## Materials and Methods

### Study sites and soil sample preparation

In NE China, the original climax vegetation in areas with typical black soils is Korean pine-broadleaf mixed forest. Historically, farmland has been gained by destroying local original vegetation including primary forests, and this process, along with the ensuing farming practices, has greatly degraded soil quality in terms of soil fertility and soil physics [36, 37]. In this paper, soils in areas of climax vegetation were selected for use as the control reference, and soils from poplar plantation forest and neighbouring farmland were sampled to evaluate the effects of afforestation on soil properties and GRSP (concentration and composition).

The referential soils were sampled in the primary forest of Korean pine-broadleaf mixed forest in Changbai National Nature Reserve: coordinates: 127°46'–128°05'N, 42°12'–42°24'E; altitude: 764–1008 m; diameter at breast height (DBH): 8.74–43.12 cm; tree height: 9.40–20.60 m; principal species: *Pinus koraiensis*, *Betula platyphylla*, *Populus spp*. (*P*. *davidiana* and *P*. *cathayana*); tree density (higher than 10 m trees): 1360 tree ha^-1^; composition ratio: pine (3 trees): birch (6 trees): poplar (1 tree). Basic data for poplar plantation forest are the following: forest age: 17–24 years; 124°19′–126°51′N, 45°08′–46°55′E; altitude: 146–400 m; DBH: 19.80–28.50 cm; tree height: 12.40–20.60 m) and neighbouring farmland soils (principal crops: corn) were selected to assess the effects of afforestation on soils. The shelterbelt poplar plantation has been afforested on farmland for over 30 years (since 1978), while the farmland has been reclaimed for over 50 years since the national reclamation of Beidahuang since 1950s. These experimental areas administratively belong to Forestry Bureau in Heilongjiang province and Changbai National Nature Reserve administration in Jilin Province, and sampling permission was granted by them. No protected species were sampled in this experiment.

Surface soil (0–30 cm) of the plantation forests (6 regions, 72 samples), farmland (6 regions, 72 samples) and primary forests (7 regions, 21 samples) was collected in NE China. The soil is typical black soil, including chernozem, phaeozem, arenosols, and cambisols, and some degraded solonetz [38]. Soil samples were collected from the soil layer (0–30 cm) with a 100-cm^3^ cutting ring. After being allowed to fully air-dry, and after small stones, distinguishable plant roots, and other debris had been excluded, samples were passed through a 0.25-mm sieve prior to laboratory analysis.

### Determination of soil fertility-related and physicochemical parameters

All 165 samples were measured for all soil parameters described herein. Soil pH was measured in a solution of 1.00 g soil sample in 5 ml deionized water using a precise pH meter, the Sartorius PB10 (Sartorius, Germany). Soil electrical conductivity (EC) was determined with an EC meter (DDS-307, Shanghai Precision Scientific Instruments Co., Ltd., China) in the same solution. Soil bulk density was calculated as the ratio between the air-dried soil mass and the soil volume (400 cm^3^, which is fixed by the soil cutting ring). SOC content was determined with the potassium dichromate volumetric method (external heating method). Total nitrogen (N) content was determined with the semi-micro Kjeldahl method. Soil total phosphorus (P) content was determined with the sodium hydroxide melt method. All the methods were from [39], and some detail descriptions can be found in [40].

### GRSP concentration determination

Extraction and determination of GRSP in the soil samples followed modified methods described by [9]. GRSP was extracted from 0.10 g of soil with 4 mL of 50 mM citrate, pH = 8.00, and autoclaved for 1 h at 121°C. In both cases, the supernatant was separated by centrifugation at 4,000 rpm for 6 min before being collected. The procedure was repeated several times (autoclaving for 30 min at 121°C) on the same sample until the reddish-brown colour typical of GRSP disappeared from the supernatant, suggesting that all extracts from a soil sample had been combined. The protein content in the crude extract was determined by Bradford assay with bovine serum albumin as the standard. The concentration of the GRSP was measured in all 165 soil samples from the farmland, poplar plantation forest, and primary forest.

### GRSP compositional characteristics determination

Purification of the GRSP was performed according to the methodology of [14]: 1.00 g soil sample was placed in 50-mL centrifuge tubes with 8 mL of 50 mM citrate extraction solvent at pH 8.00. The sample was oscillated for half a minute to ensure thorough mixing and then autoclaved at 121°C for 60 min. Next, the sample was centrifuged at 4,000 rpm for 15 min, and the supernatant was removed to centrifuge cups. For the residual sample in 50-mL centrifuge tubes, the same processes (added 8 mL citrate extraction solvent, oscillated for half a minute, autoclaved at 121°C for 60 min, centrifuged at 4,000 rpm for 15 min, and the supernatant was removed to centrifuge cups) were repeated until the supernatant no longer showed the typical red brown colouration. The glomalin extract was then precipitated by titrating to a pH of 2.10 with 0.10 M hydrochloric acid, centrifuging at 4,000 rpm for 15 min, and incubating the extraction samples on ice for 60 min. The pellet at the bottom of the centrifuge tube was resolubilized in 0.10 M sodium hydroxide until it was completely reconstituted, and the samples dialyzed against deionized water for 60 h (dialysis bag, DW = 8,000–14,000 Da, Scientific Research Special, USA). After dialysis, the purified dialyzate was centrifuged at 10,000 rpm for 10 min to remove any extraneous particles. The supernatant was then immediately freeze-dried with a vacuum freeze drier (Scientz-10N, Ningbo Scientz Biotechnology Co., Ltd., China). For this part of the study, 19 composite soil samples from different regions were used in the experiments (6 from different farmland regions, 6 from different poplar plantation regions, and 7 from different primary forest regions).

### GRSP fluorescent characteristics from the 3-D fluorescence measurement

The methodology for obtaining the fluorescence spectrometer measurements is described in [15]. For this measurement, 1.00 mg of a freeze-dried GRSP sample (an electronic balance with 0.01 mg precision, DV215CD, Ohaus Instrument (Shanghai) Co. Ltd, America) was placed in a 10-ml centrifuge tube, dissolved in 1 ml of 0.10 M sodium hydroxide solution, and diluted 5-fold. A Hitachi F-7000 fluorescence spectrometer (Hitachi High Technologies, Tokyo, Japan) with a 700-voltage xenon lamp at room temperature (20 ± 2°C) was used to acquire readings. These readings were collected in ratio mode (S/R) (the default mode of the F-7000 fluorescence spectrometer), using a scanning speed of 2400 nm/min. The Excitation-Emission Matrix (EEM) spectra were scanned at a range of 210–550 nm for excitation, and 280–650 nm for emission. The bandpass widths were 5 nm for both excitation and emission. The range of the different excitation/emission wavelengths of the fluorescence spectra classified the dissolved organic matter in GRSP into 8 main fluorescent substances (appearing at frequencies between 60% and 100%) in accordance with the reference study [41] and with identification support from Microspheres Online (http://microspheres.us/microsphere–basics/fluorochromes–excitation–emission–wavelengths/248.html).

The PARAFAC analysis of the EEM spectra was conducted according to [42]. All EEM spectra data made up a three-way array X with I × J × K. The three-way array X was able to decompose into trilinear terms and a residual array according to the following:

$$X_{\text{ijk}}=\sum_{f}^{F}c_{if}b_{jf}a_{kf}\;+\;\sigma_{ijk}\;i=1,\ldots ,I;\;j=1,\ldots ,J;\;k=1,\ldots ,K$$

where X_ijk_ was the fluorescence intensity of the sample i at the emission wavelength j and excitation wavelength k; F was the number of components; c_if_ was directly proportional to the concentration of the fluorophore component f in the sample i; b_if_ and a_kf_ were estimates of the emission and excitation spectra, respectively, for the fluorophore component f; and σ_ijk_ was the residual term, which represented the variability not accounted for by the model [43].

EEM data were combined into a single Excel file; this file was imported into Matlab 7.0, and then, PARAFAC modelling was carried out using the n-way toolbox for Matlab 7.0 found at http://www.models.kvl.dk/source/nwaytoolbox/ [44]. In order to present EEM data more quantitatively, multi-way data analysis was used to process the 3-D EEM fluorescence data.

### GRSP elemental characteristics from the XPS analysis

A K-Alpha spectrometer equipped with a concentric hemispherical analyser in the standard configuration (Thermo Scientific, USA) was used in this analysis with the method revised from [45]. After the preparation of control and the freeze-dried GRSP samples, the soil colloidal solution was dropped on a clean, highly ordered pyrolithic graphite (HOPG) surface and then air-dried before examination. The vacuum system consisted of a turbomolecular pump and a titanium sublimation pump. The residual pressure before the analysis was lower than 10^−7^ Pa. The X-ray source was AlKα, and was run at 30 mA and 80 kV. The incident angle was 49.1°, and the emission angle was 0° with respect to the sample’s normal surface. All spectra were obtained in digital mode. The wide-scan spectra were acquired from 1000 eV to 0 eV. Sample charging was corrected by comparing all binding energies to the adventitious carbon at 285 eV. Detailed spectra were processed using Casa XPS software (V2.3.12, Casa Software Ltd., UK). An iterated Shirley-Sherwood background subtraction was applied before peak fitting using a nonlinear least squares algorithm. The atomic concentration of the elements was calculated using the Casa XPS software [33].

### GRSP functional groups from infrared spectrometer

A total of 2.00 mg freeze-dried GRSP samples were placed in a mortar and 0.20 g potassium bromide powder was added. These were fully ground and pressed into a wafer. Functional groups were determined with an IRAffinity-1 infrared spectrometer model (SHIMADZU, Japan) with a spectral range of 4000–500 cm^–1^. For each peak in the spectrum, the absorption peak area can semi-quantitatively reflect the concentration of the functional group that matches the peak. The matching of functional groups with peak wave numbers was described in [46]. Seven kinds of functional groups in GRSP were found by using the IR schematic diagram with partition method for functional groups of GRSP that is provided in [15].

### Data analysis

An analysis of variance (ANOVA) with least significance difference (LSD) pairwise comparison was used to identify the various land use-related variations in soil properties and GRSP (amount and composition). Regression analysis was used to find linear relations between soil properties and GRSP (amount and composition). The analysis of the data collected via the abovementioned techniques was performed by SPSS 17.0 (SPSS, USA).

## Results

### Correlation analysis between GRSP (amount and composition) and soil fertility parameters

A total of 78 pairs of correlations between soil fertility-related parameters and GRSP composition were checked, and 27 pairs of them (related with SOC and total N in the soil) were statistically significant (p < 0.05) (Table 1). SOC and total N were also positively correlated with the relative contents of tryptophan-like and fulvic acid-like fluorescence; O–H bending of structural OH; elements of Al, O, and Si in GRSP (p < 0.05), but were negatively correlated with the relative occurrence of stretching of aliphatic C–H, stretching of C–O, O–H bending of –COOH; elements of C, Ca, and Na in GRSP (p < 0.05) (Table 1).

**Table 1:** Significant correlations between soil fertility-related parameters and GRSP composition (p < 0.05). Non-significant correlations are not displayed (p > 0.05).

GRSP composition	SOC (g/kg)		Total N (g/kg)	
	Equation	R^2^	Equation	R^2^
**Correlations between fluorescent substances and soil fertility-related parameters**				
**Tryptophan-like in GRSP**	y = 0.24x+0.52	0.49	y = 3.52x–0.15	0.39
**Fulvic acid-like in GRSP**	y = 0.88x–0.97	0.42	y = 11.92x–1.85	0.30
**Humic acid-like in GRSP**	y = 0.63x+22.81	0.31	–	–
**Nile red-like in GRSP**	–	–	y = –2.62x+16.09	0.26
**Correlations between functional groups and soil fertility-related parameters**				
**Stretching of aliphatic C–H in GRSP**	y = –3.35x+309.09	0.42	y = –58.24x+337.90	0.49
**Stretching of C–O and O–H bending of –COOH in GRSP**	y = –1.92x+112.78	0.53	y = –31.01x+124.60	0.54
**Stretching of C = O, asymmetric COO–in GRSP**	y = –6.12x+2047.80	0.26	y = 98.57x+2084.90	0.26
**O–H bending of structural OH in GRSP**	y = 1.35x+8.91	0.57	y = 23.02x–1.81	0.64
**Correlations between elements and soil fertility-related parameters**				
**Al in GRSP**	y = 0.09x+0.53	0.50	y = 1.41x+0.02	0.49
**O in GRSP**	y = 0.31x+28.02	0.50	y = 5.24x+25.66	0.55
**Si in GRSP**	y = 0.19x+0.88	0.56	y = 2.87x+0.17	0.48
**C in GRSP**	y = –0.51x+57.56	0.44	y = –8.37x+61.02	0.46
**Ca in GRSP**	y = –0.01x+0.77	0.52	y = –0.13x+0.75	0.30
**N in GRSP**	y = –0.06x+5.15	0.59	–	–
**Na in GRSP**	y = –0.03x+3.82	0.65	y = –0.33x+3.77	0.37

**Fig 1:**
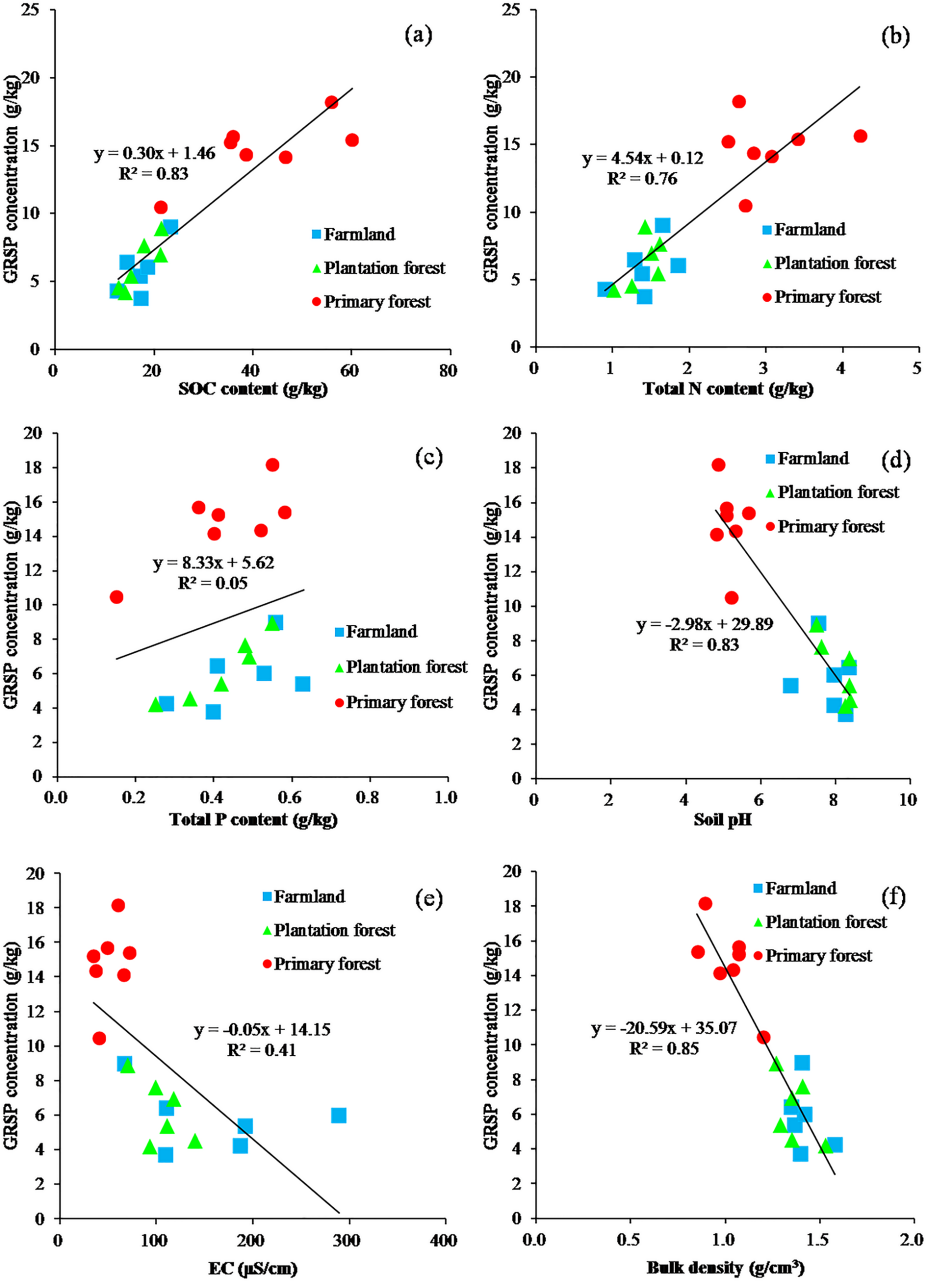
Correlations between GRSP concentration and soil properties. Correlations between GRSP concentration and: (a) SOC content (p<0.05); (b) total N content (p<0.05); (c) total P content (p>0.05); (d) soil pH (p<0.05); (e) EC (p<0.05); (f) bulk density (p<0.05). N = 19 = Primary forest (7) + Plantation forest (6) + Farmland (6).

In addition to the GRSP compositional characteristics, 2 marked correlations were observed between soil fertility-related parameters and GRSP concentration (Fig 1): SOC (R^2^ = 0.83) and total N (R^2^ = 0.76) were positively related with GRSP concentration (p < 0.05). Also, there was a positive correlation between total P and GRSP concentration (p > 0.05) (Fig 1).

### Correlations between GRSP (amount and composition) and soil physicochemical properties

We checked 78 pairs of correlations between soil physicochemical properties and GRSP compositions, and 38 pairs of significant correlations were observed (Table 2). Soil pH was negatively correlated with the relative contents of tryptophan-like, fulvic acid-like, and humic acid-like fluorescence; O–H bending of structural OH; elements of Al, O, and Si in GRSP (p < 0.05). The relative contents of nitrobenzoxadidole-like fluorescence; stretching of the O–H, N–H, aromatic C–H, aliphatic C–H, C = O, asymmetric COO–, C–O and O–H bending of the –COOH; elements of C, Ca, N, and Na were positively correlated with soil pH (p < 0.05). The R^2^ of these factors ranged from 0.25 to 0.90, and correlations with R^2^ > 0.80 included soil pH and the relative contents of stretching of C–O and O–H bending of –COOH in GRSP (positive correlations, R^2^ = 0.85); soil pH and the relative content of O–H bending of structural OH in GRSP (negative correlations, R^2^ = 0.90); and soil pH and Al content in GRSP (negative correlations, R^2^ = 0.83) (Table 2).

**Table 2:** Significant correlations between soil physicochemical parameters and GRSP composition (p < 0.05). Non-significant correlations are not displayed (p > 0.05).

GRSP compositions	Soil pH		Bulk density (g/cm^3^)		EC (μS/cm)	
	Equation	R^2^	Equation	R^2^	Equation	R^2^
**Correlations between fluorescent substances and soil physicochemical properties**						
**Tryptophan-like in GRSP**	y = –2.40x+23.57	0.46	y = –16.62x+27.80	0.47	–	–
**Fulvic acid-like in GRSP**	y = –9.12x+85.23	0.44	y = –60.34x+97.80	0.42	–	–
**Humic acid-like in GRSP**	y = –5.84x+79.76	0.26	y = –40.60x+90.30	0.27	–	–
**Nitrobenzoxadidole-like in GRSP**	y = 2.99x+20.22	0.27	–	–	–	–
**Correlations between functional groups and soil physicochemical properties**						
**Stretching of O–H, N–H, aromatic C–H in GRSP**	y = 195.74x+6420.00	0.30	–	–	–	–
**Stretching of aliphatic C–H in GRSP**	y = 39.92x–55.73	0.59	y = 241.47x–82.16	0.46	–	–
**Stretching of C = O, asymmetric COO–in GRSP**	y = 75.95x+1360.70	0.39	y = 452.99x+1318.40	0.24	–	–
**Stretching of C–O and O–H bending of –COOH in GRSP**	y = 24.44x–107.01	0.85	y = 129.19x–99.84	0.51	y = 0.34x+27.27	0.32
**O–H bending of structural OH in GRSP**	y = –17.01x+162.30	0.90	y = –100.27x+70.27	0.66	y = –0.26x+70.89	0.40
**Correlations between elements and soil physicochemical properties**						
**Al in GRSP**	y = –1.14x+10.77	0.83	y = –6.40x+10.89	0.56	y = –0.02x+4.62	0.36
**O in GRSP**	y = –3.69x+61.76	0.70	y = –21.44x+63.10	0.50	–	–
**Si in GRSP**	y = –2.26x+21.59	0.77	y = –13.51x+22.88	0.58	y = –0.03x+9.53	0.36
**Mg in GRSP**	–	–	–	–	y = –0.002x+0.61	0.22
**Ca in GRSP**	y = 0.09x–0.15	0.40	y = 0.67x–0.35	0.44	y = 0.002x+0.31	0.27
**C in GRSP**	y = 6.59x–1.41	0.73	y = 36.96x–2.18	0.49	y = 0.11x+32.70	0.41
**N in GRSP**	y = 0.17x+1.96	0.25	y = 4.04x–1.52	0.55	y = 0.01x+2.46	0.35
**Na in GRSP**	y = 0.69x–1.24	0.76	y = 1.78x+0.88	0.61	–	–

There were negative correlations between soil bulk density and the relative contents of tryptophan-like, fulvic acid-like, humic acid-like fluorescence; O–H bending of structural OH; elements of Al, O, and Si in GRSP (p < 0.05), while positive correlations were observed between bulk density and the relative contents of stretching of aliphatic C–H, C = O, asymmetric COO–, C–O; O–H bending of –COOH; elements of C, Ca, N, and Na in GRSP (p < 0.05) (Table 2). The R^2^ ranged from 0.24 to 0.66, and the closest correlations were found between soil bulk density and the relative content of O–H bending of the structural OH in GRSP (negative correlations, R^2^ = 0.66), and soil bulk density and Na content in GRSP (positive correlations, R^2^ = 0.61) (Table 2).

Compared with soil pH and soil bulk density, fewer significant correlations between EC and GRSP compositional characteristics were observed (Table 2). No correlations were found between fluorescent substances in GRSP and EC (p > 0.05). The relative contents of two functional groups (stretching of C–O and O–H bending of –COOH, O–H bending of structural OH) and GRSP elemental composition parameters (Al, C, Si, Ca, Mg, and N) were significantly correlated with EC (p < 0.05). The R^2^ for these (0.22 to 0.41) was also much lower than those for soil pH and soil bulk density (Table 2).

Soil physicochemical parameters (soil pH, bulk density and EC) were negatively correlated with GRSP concentration (p < 0.05). R^2^ of bulk density (0.85) and soil pH (0.83) was greater than that of EC (0.41) in linear correlations (Fig 1).

### Correlations analysis between GRSP amount and composition

We checked 26 pairs of correlations between GRSP concentration and composition, and 15 pairs of significant correlations were observed (Table 3). GRSP concentration showed positive correlations with the relative contents of tryptophan-like, fulvic acid-like, and humic acid-like fluorescence; O–H bending of structural OH; elements of Al, O, and Si (p < 0.05), while negative correlations were observed between GRSP concentration and the relative contents of Nile red-like fluorescence; stretching of aliphatic C–H, C = O, asymmetric COO–, and C–O; O–H bending of –COOH; elements of C, Ca, N, and Na. The R^2^ for these linear correlations ranged from 0.21 to 0.78. A comparison of the value of R^2^ suggested that the relative contents of tryptophan-like fluorescence, O–H bending of structural OH, and Al element were tightly correlated with GRSP concentration (Table 3).

**Table 3:** Significant correlations between GRSP concentration and composition (p < 0.05). Non-significant correlations are not displayed (p > 0.05).

GRSP compositions	GRSP concentration (g/kg)	
	Equation	R^2^
**Correlations between fluorescent substances and GRSP concentration**		
**Tryptophan-like in GRSP**	y = 0.81x–0.59	0.57
**Fulvic acid-like in GRSP**	y = 2.98x–5.38	0.51
**Humic acid-like in GRSP**	y = 2.02x+20.71	0.33
**Nile red-like in GRSP**	y = –0.44x+15.14	0.26
**Correlations between functional groups and GRSP concentration**		
**Stretching of aliphatic C–H in GRSP**	y = –11.84x+330.18	0.55
**Stretching of C = O, asymmetric COO–in GRSP**	y = –17.28x+2046.30	0.21
**Stretching of C–O and O–H bending of –COOH in GRSP**	y = –6.76x+124.71	0.69
**O–H bending of structural OH in GRSP**	y = 4.87x–0.50	0.78
**Correlations between elements and GRSP concentration**		
**Al in GRSP**	y = 0.33x–0.21	0.74
**O in GRSP**	y = 1.11x+25.94	0.67
**Si in GRSP**	y = 0.69x–0.37	0.74
**Ca in GRSP**	y = –0.03x+0.77	0.46
**C in GRSP**	y = –1.93x+62.00	0.67
**N in GRSP**	y = –0.21x+5.46	0.72
**Na in GRSP**	y = –0.07x+3.77	0.47

### Variations of soil properties in different land uses

SOC content (41.94 g/kg) and total N content (3.05 g/kg) in primary forest were 2.40-fold and 2.20-fold greater than in farmland and plantation forest (p < 0.05), and no differences were found between farmland and plantation forest (p > 0.05). Total P content in the farmland had the highest value (0.47 g/kg), and the lowest value (0.42 g/kg) was in primary forest and plantation forest; however, there was no significant variation among the 3 land-use types (p > 0.05) (Table 4).

**Table 4:** Variations of GRSP amount, composition and soil properties in different land-uses.

Index	Primary forest	Plantation forest	Farmland
**GRSP amount (mean and their statistical significance)**			
**GRSP concentration (g/kg)**	14.78a	6.29b	5.77b
**GRSP compositions (mean and their statistical significance)**			
**Elements**			
**Al in GRSP**	4.79a	1.28b	2.17b
**C in GRSP**	33.36b	52.19a	48.69a
**K in GRSP**	1.86a	2.67a	1.17a
**P in GRSP**	0.38a	0.30a	0.28a
**O in GRSP**	42.60a	31.45b	33.56b
**Si in GRSP**	9.69a	3.01b	4.50b
**Ca in GRSP**	0.30b	0.55a	0.65a
**Fe in GRSP**	1.36a	0.92a	1.03a
**Mg in GRSP**	0.51a	0.35a	0.28a
**N in GRSP**	2.39b	4.31a	4.15a
**Na in GRSP**	2.78b	3.20ab	3.42a
**Functional groups**			
**Stretching of O–H, N–H, aromatic C–H in GRSP**	7415.66a	8050.88a	7917.38a
**Stretching of aliphatic C–H in GRSP**	157.33b	258.80a	256.12a
**Stretching of C = O, asymmetric COO–in GRSP**	1746.96b	2039.90a	1895.38ab
**Stretching of symmetric COO–and bending of C–H in GRSP**	417.29b	500.88a	459.20ab
**Stretching of C–O and O–H bending of –COOH in GRSP**	20.94b	95.12a	77.17a
**Stretching of Si–O–Si and polysaccharide C–O in GRSP**	1283.29a	1277.33a	1222.57a
**O–H bending of structural OH in GRSP in GRSP**	75.67a	25.65b	27.18b
**Fluorescent substances**			
**Tryptophan-like in GRSP**	11.48a	3.55b	5.04b
**Fulvic acid-like in GRSP**	39.57a	11.15b	12.89b
**Humic acid-like in GRSP**	51.57a	27.30a	37.21a
**Nitrobenzoxadidole-like in GRSP**	35.57a	45.89a	42.19a
**Tyrosine-like in GRSP**	3.70a	1.79a	1.94a
**Nile red-like in GRSP**	5.86a	12.20a	10.84a
**Soluble microbial byproduct-like in GRSP**	7.40a	3.72a	4.72a
**Calcofluor white-like in GRSP**	20.69a	33.64a	33.96a
**Soil fertility-related parameters (mean and their statistical significance)**			
**SOC (g/kg)**	41.94a	17.2b	17.43b
**Total N (g/kg)**	3.05a	1.39b	1.41b
**Total P (g/kg)**	0.42a	0.42a	0.47a
**Soil physicochemical parameters (mean and their statistical significance)**			
**Soil pH**	5.14c	8.08a	7.83b
**EC (μS/cm)**	51.4b	105.22b	159.85a
**Bulk density (g/cm** ^**3**^ **)**	1.01c	1.37b	1.42a

Note: Lowercase letters stand for significance of 0.05 (and that these comparisons are across each row).

Soil pH, EC, and bulk density differed significantly among the 3 land uses (p < 0.05) (Table 4). Soil pH in primary forest was as low as 5.14, which was significantly lower than in plantation forest (8.08) and farmland (7.83) (p < 0.05). In addition, the soil pH of the plantation forest was significantly higher than in farmland (p < 0.05). EC in farmland peaked at 159.85 μS/cm, about 1.50-fold and 3-fold greater than in plantation forest and primary forest, respectively (p < 0.05). The value for soil bulk density in primary forest was the lowest (1.01 g/cm^3^) and was significantly lower than in plantation forest and farmland (p < 0.05); furthermore, there was a significant difference in this value between plantation forest and farmland (p < 0.05) (Table 4).

### Variations of GRSP amount and compositional characteristics (fluorescent substances, functional groups, and elemental characteristics) in different land uses

The concentration of GRSP in primary forest (14.78 g/kg) was significantly higher than in plantation forest (6.29 g/kg) and farmland (5.77 g/kg) (p < 0.05). The 9% increase in plantation forest compared with farmland was not statistically significant (p > 0.05) (Table 4).

In total, 42 types of fluorescent substances were found in GRSP from farmland, 33 substances were observed in GRSP from plantation forest, and 30 substances were observed in GRSP from primary forests (Table 5). Of all the studied samples, 4 fluorescent substances were found in all (100% occurrence), and these were tryptophan-like, fulvic acid-like, humic acid-like, and nitrobenzoxadidole-like fluorescence. Two substances, tyrosine-like and Nile red-like fluorescence were observed in more than 80% of all samples. In addition, 2 fluorescent substances (soluble microbial byproduct-like and calcofluor white-like fluorescence) occurred with a frequency of 60%–80%. In general, for these 8 main fluorescent substances (> 60% occurrence), there was a tendency for the differences between farmland and plantation forest to be much smaller than the differences between those two land-use types and primary forest (Table 5). Relative content of tryptophan-like fluorescence in the primary forest was 2.28-fold and 3.23-fold higher than in farmland and plantation forest (p < 0.05), respectively, and relative content of fulvic acid-like fluorescence was 3.07-fold and 3.55-fold higher than farmland and plantation forest (p < 0.05), respectively. However, there was no difference between farmland and plantation forest in these 2 parameters (p > 0.05). All other fluorescent substances showed no difference among the 3 land uses (p > 0.05). In addition, compared with farmland and plantation forest, primary forest had the highest value for humic acid-like, tyrosine-like and soluble microbial byproduct-like fluorescence, but the lowest value for nitrobenzoxadidole-like, Nile red-like and calcofluor white-like fluorescence (Table 4).

**Table 5:** 49 fluorescent substances were observed in different land-uses with parallel factor analysis.

No.	EX/EM (nm)	Possible fluorescent substances	Possible molecular formula	Frequency for observation of the substances				Percentage (%)
				Plantation forest (No)	Farmland (No)	Primary forest (No)	Total (No)	
**1**	220-250/330-380	Tryptophan-like	C_11_H_12_N_2_O_2_	6	6	7	19	100
**2**	220-250/380-480	Fulvic acid-like	C_135_H_182_O_95_N_5_S_2_	6	6	7	19	100
**3**	250-420/380-520	Humic acid-like	C_187_H_186_O_89_N_9_S_1_	6	6	7	19	100
**4**	460-470/510-650	(Cl-) Nitrobenzoxadidole-like	C_6_H_2_ClN_3_O_3_	6	6	7	19	100
**5**	220-250/280-330	Tyrosine-like	C_9_H_11_NO_3_	5	5	6	16	84.21
**6**	515-530/525-605	Nile red-like	C_20_H_18_N_2_O_2_	6	5	5	16	84.21
**7**	250-360/280-380	Soluble microbial byproduct-like	-	6	3	3	12	63.16
**8**	440/500-520	Calcofluor white-like	C_40_H_42_N_12_O_10_S_2_·2Na	4	4	4	12	63.16
**9**	450-490/515	Aurophosphine	C_16_H_18_N_3_Cl	3	3	3	9	47.37
**10**	470/540	Astrazon orange R	C_28_H_27_ClN_2_	3	2	4	9	47.37
**11**	540-590/560-650	Pyronine B	C_21_H_27_ClN_2_O	3	2	2	7	36.84
**12**	523-557/595	Rhodamine B 200	C_28_H_31_ClN_2_O_3_	3	1	2	6	31.58
**13**	540/550-600	Rose bengal	C_20_H_4_Cl_4_I_4_O_5_	2	2	2	6	31.58
**14**	440/530	Sevron orange	C_22_H_23_ClN_2_	1	2	3	6	31.58
**15**	365/395	9-(Bis amino-phenyl oxadiazole)	C_14_H_12_N_4_O	1	4	0	5	26.32
**16**	355-425/460	Acriflavin-like	C_27_H_25_ClN_6_	2	1	1	4	21.05
**17**	380-415/520-530	5-Hydroxy-tryptamine	C_10_H_12_N_2_O	1	3	0	4	21.05
**18**	370-385/477-484	Thiolyte	C_10_H_11_BrN_2_O_2_	1	2	1	4	21.05
**19**	465/535	NBD chloride-like	C_6_H_2_ClN_3_O_3_	3	0	1	4	21.05
**20**	430/520	Brilliant sulpho-flavin FF	C_19_H_14_ N_2_ O_5_S·Na	1	2	1	4	21.05
**21**	470/550	Acridine yellow	C_15_H_15_N_3_	1	2	1	4	21.05
**22**	500/585	Astrazon brilliant red 4G	C_23_H_26_ClN_3_	1	2	1	4	21.05
**23**	365/420-430	True blue	C_20_H_18_Cl_2_N_4_O_2_	2	1	0	3	15.79
**24**	340-380/430	Diamino naphthyl-sulphonic acid	C_16_H_13_N_5_O_5_S	1	1	1	3	15.79
**25**	310-370/520	Dimethylamino-5-sulphonic acid	C_12_H_13_NO_3_S	2	1	0	3	15.79
**26**	465/565	Phosphine	PH_3_	1	0	2	3	15.79
**27**	530-560/580	Alizarin complexon	C_19_H_15_NO_8_	1	1	1	3	15.79
**28**	535-553/605	Pontachrome blue black BB-like	C_20_H_13_N_2_O_5_S·Na	1	1	1	3	15.79
**29**	530/590	Sevron brilliant red B	C_24_H_15_N_3_Na_2_O_7_S_3_	2	1	0	3	15.79
**30**	510/580	Thiazine red R	C_24_H_15_N_3_O_7_S_3_·2Na	0	2	0	2	10.53
**31**	365/495	Nuclear yellow	C_25_H_25_N_7_O_2_S·3HCl	1	1	0	2	10.53
**32**	365/460	Stilbene isothio sulphonic acid	C_14_H_12_N_2_O_8_S_2_	1	1	0	2	10.53
**33**	375/430	C.I. Fluorescent brightener 41-like	C_15_H_11_NS	0	1	1	2	10.53
**34**	340/490-520	Dopamine	C_8_H_11_NO_2_	0	1	1	2	10.53
**35**	450/530	7-Nitrobenz-2-Oxa-1,3-Diazole (NBD) Amine	C_38_H_68_N_4_O_3_	0	0	2	2	10.53
**36**	430/550	Berberine sulphate	C_40_H_36_N_2_O_8_O_4_S	0	1	1	2	10.53
**37**	370/440	Calcofluor RW	C_14_H_17_NO_2_	1	0	0	1	5.26
**38**	370/430	Leucophor PAF	C_40_H_40_N_12_Na_4_O_16_S_4_	1	0	0	1	5.26
**39**	395/465	Leucophor WS	C_14_H_17_NO_2_	0	1	0	1	5.26
**40**	350/405-482	Indo-1	C_32_H_31_N_3_O_12_	0	1	0	1	5.26
**41**	360/465	7-Hydroxy-4-methylcoumarin	C_10_H_8_O_3_	0	1	0	1	5.26
**42**	360/430	Intrawhite CF	C_34_H_28_N_10_Na_2_O_8_S_2_	0	1	0	1	5.26
**43**	450/580	Aurophosphine G	C_16_H_18_ClN_3_	0	1	0	1	5.26
**44**	460/550	Auramine	C_7_H_21_N_3_	0	1	0	1	5.26
**45**	430/540	Euchrysin	C_16_H_18_ClN_3_	0	1	0	1	5.26
**46**	470/565	Rhodamine 5 GLD	C_27_H_29_N_2_O_3_Cl	0	1	0	1	5.26
**47**	430/535	Lucifer yellow	C_13_H_10_Li_2_N_4_O_9_S_2_	0	1	0	1	5.26
**48**	540/580	Rhodamine B	C_28_H_31_ClN_2_O_3_	0	0	1	1	5.26
**49**	520/595	Astrazon red 6B	C_24_H_30_C_l2_N_2_	0	0	1	1	5.26
**Total observations in each land-use**				33	42	30		

In the case of GRSP functional groups, primary forest had much lower values in 2 functional groups (stretching of the aliphatic C–H (approximately 1.60-fold) and stretching of the C–O and O–H bending of the –COOH (3.70–4.50-fold)), but much lower O–H bending of structural OH (approximately 35%) compared with farmland and plantation forest (p < 0.05). However, there were no differences among these 3 functional groups between farmland and plantation forest (p > 0.05) (Table 4). Plantation forest showed significantly higher stretching of C = O, asymmetric COO–(1.16-fold), and stretching of symmetric COO–and bending of C–H (1.23-fold) than those in primary forest (p < 0.05), and intermediate values for these characters were found in farmland with no significant difference between farmland and primary and plantation forest (p > 0.05) (Table 4). There were no differences among the 3 land uses in the stretching of O–H, N–H, aromatic C–H, Si–O–Si, and polysaccharide C–O (p > 0.05) (Table 4).

With the relative contents of elements in GRSP, significant differences in the 3 land uses were found in all investigated elements except for K, P, Fe and Mg (Table 4). Much higher values of Al (2.20–3.80-fold), O (approximately 1.30-fold), and Si (2.20–3.30-fold), and much lower values of C (64%–68%), Ca (approximately 50%), and N (approximately 55%) in GRSP were found in primary forest than those in plantation forest and farmland (p < 0.05). However, there were no differences in these 6 elements between farmland and plantation forest (p > 0.05). Na content in GRSP from farmland was 1.23-fold higher than in primary forest (p < 0.05), and the Na content from both land-use types did not differ from plantation forest (Table 4).

## Discussion

### GRSP composition is more complex than previously reported

GRSP composition is more complex than has been previously reported. GRSP is an unusual substance that has been difficult to analyze biochemically due to its recalcitrance and complexity [47]. In the past, some advanced techniques and methods have been applied in studying GRSP composition. Lectin-binding capability and high performance capillary electrophoresis data have shown glomalin to be a glycoprotein with a major asparagine-linked (N-linked) chain of carbohydrates [9]. Gel electrophoresis and NMR spectroscopy have suggested that GRSP is polymeric with iron [12, 48]. Nichols [49] reported that GRSP was a compound tightly bound with iron, and composed by organic matter, amino acids and carbohydrates. In general, past and current reports indicated that GRSP composition consists of a singly glycosylated protein, aromatics, carboxyls, carbohydrates, lipids, humics, amino acids, and some elements (e.g. C, N, O, H, Fe, Cr, Cu, etc.) [12–14, 16, 17, 48]. In our study, 8 fluorescent substances, 7 kinds of functional groups and 11 kinds of elements were also observed in GRSP composition. Our study further complements GRSP composition, indicates that GRSP composition is more complicated than originally expected, and suggests the need for further study.

Many researchers studied either GRSP amount or GRSP composition, but few reported the relationships between them and our data suggested that the GRSP amount in soil is dependent of its compositional features. Our results showed that the GRSP concentrations were negatively correlated with the relative contents of Nile red-like fluorescence; functional groups of aliphatic C–H, C = O, C–O, asymmetric COO–and O–H bending of –COOH; elements of C, Ca, N, Na. It has been suggested that glomalin is an artifact of the extraction processes [14]. The glomalin compositional variations observed herein indicate that the extraction method should be further refined for a more exact and reliable procedure.

### Marked correlations between GRSP (amount and composition) and soil properties indicate the important role of GRSP in soil

Although some studies tried to relate GRSP concentration to abiotic and biotic factors [50, 51], few papers have focused on the relationships between GRSP composition and soil properties. Our results suggested the significant effect of GRSP in associating with soil properties were not only in its concentration, but also its compositional characteristics.

SOC content and N content (soil fertility-related properties) have great impacts on both GRSP concentration and composition (main functional groups, fluorescent substances, and elements). Consistent with our results, some previous studies also reported that evident correlations between GRSP concentration and SOC content (R^2^ = 0.73, 0.79) [52, 53], total N content (R^2^ = 0.89, 0.63) [54, 55], available N content (R^2^ = 0.62, 0.68) [56, 57]. However, information on the factors affecting GRSP composition is very scarce and uncertain. The present study shows that some of the GRSP composition had relations with SOC and total N in soil, including tryptophan-like and fulvic acid-like fluorescence; functional group of O–H bending of structural OH; elements of Al, O, Si (Table 1). Therefore, our results indicate potentially important roles of GRSP (amount and composition) in contributing to soil fertility (SOC and N). These relationships may help us to understand the underlying mechanism of improving soil quality.

Additionally, there was no significant correlation between GRSP (concentration and composition) and total P content (soil fertility-related property). Supporting our results, previous study also reported that GRSP concentration may not depend on soil P content [55]. Similarly, according to [58], GRSP concentration was positively correlated with SOC, negatively with polyphenol oxidase activity, but not with hyphal length or phosphatase activity. These results indicate that AM-mediated production of GRSP and relevant soil enzyme activities may not depend on external P concentrations. By contrast, Dai et al. [59] suggested that long-term P-containing fertilization significantly increased SOC content, AM fungal population size, as well as GRSP concentration. Function of soil fungi in improving plant P nutrient supply have been well evaluated in plant nutrient uptake [60], soil collide surface alternation [33] as well as extracellular enzymatic influences [61]. The non-association with GRSP indicates that only limited amount of P was retained in the fungal hyphae residues after decomposition (GRSP), and this may make hyphae more efficiently transport P from soil into plants without more storage in this own biomass and decomposed residues.

Soil pH is a key factor in the observed differences both in GRSP amount and composition. Past and present reports have indicated that neutral or slightly acidic soil is suitable for the accumulation of GRSP, possibly from the well-developed plant roots and various soil fungi [19, 54, 55]. Besides the correlation between soil pH and GRSP concentration, there were 7 measures of GRSP composition (tryptophan-like, fulvic acid-like, humic acid-like, O–H bending of structural OH, Al, O, Si) that showed significant negative correlations with soil pH.

Significant negative correlations between soil bulk density and GRSP concentration have been observed, indicates that their effect in improving soil structure [15, 54, 62, 63]. Our studies showed that both GRSP amount and GRSP composition are important for soil bulk density changes and EC variations. Our study revealed that 14 measures of GRSP composition were significantly related with soil bulk density. In addition, increasing GRSP concentration accompanied with decreasing soil EC, and 8 measures of GRSP composition (functional groups and elements) were closely related with soil EC (Table 2). To our knowledge, none of the relevant studies have clearly indicated the correlations between GRSP (concentration and composition) and soil EC.

### Thirty years poplar afforestation on farmland improved soil physicochemical properties, but the properties still differ significantly from those in primary forest: implications

NE China is the most important grain commodity region with largest scale of land reclamation in history and is presently experiencing serious soil degradation [36, 37]. Despite the extensive literature on the effect of afforestation of formerly arable land on soil properties [64], the time period for recovery and measures for improving forest management are still in debates for a specific region [64, 65]. In this paper, 30 years of growth of poplar afforestation can improve degraded farmland soil quality in soil bulk density (decreased by 4%) and EC (decreased by 34%). However, when compared with levels of the investigated soil characteristics in climax primary forests, the afforestation-induced changes in the soil were still small. Moreover, 30 years poplar afforestation on degraded farmland is not enough to recover GRSP to the level of non-degraded primary forest (Table 4).

The large differences in values observed between plantation forest and climax primary forest, as well as the relatively small differences observed between plantation forest and neighbouring farmland (Table 4), imply that a longer term is needed to recover soil properties with afforestation. Several implications for improving soil quality can be derived from this paper.

Firstly, the RFTF programme has implemented for 16 years (from 1999), and the second-term is just initiated in 2015. Great progress has been reported in biomass improvement, wildlife recovery, and environmental regulations [66]. However, a longer duration (over 30 years) is needed from RFTF programme for rehabilitating the underground functions of soil, especially the GRSP-related soil fungi improvement.

Secondly, close-to-nature forest management combined with mixed forest plantation have been recognized as the effective way to improve environmental ecology and create the best living community, inevitable trends in afforestation in the future, and it is also a good way to rehabilitate soil quality, as shown in the data in this paper. Natural poplar-birch forest, a common forest type in NE China, has similar soil properties to Korean pine-broadleaf forests in this region [67]. Currently, poplar plantation as farm shelterbelt, which is significant to control desertification, improve farm production, establish new ecological balance, and develop regional economy [68]. However, poplar plantation showed less soil improvement than natural forests (as shown in this paper). Compared with pure forest, some close-to-nature forest management, such as artificial mixed forest could increase soil improvements [69]. In local afforestation practices, some species naturally mixed with poplar natural forests, such as Betula platyphylla, Pinus koraiensis, Fraxinus mandshurica, Quercus mongolica, Acer triflorum are welcomed mixed species [67, 70]. Consistent with our suggestion, some studies also suggested close-to-nature forest management had a large impact on improving forest soil quality [71, 72].

## Conclusion

GRSP consists of 49 fluorescent substances, 7 measures of functional groups, and several elements. Our results highlight that GRSP composition and characteristics are more complex than previously reported. GRSP concentration was closely correlated with soil pH, bulk density, EC, SOC and total N, and close relations between soil properties and some aspects of GRSP composition were also observed. Afforestation on farmland decreased EC and bulk density. However, the GRSP concentration and composition were not different between farmland and plantation forest.
